# The Plasma Glucose Threshold Values Associated with Adverse Pregnancy Outcomes Among Asian Indian Pregnant Women: MAASTHI Birth Cohort Analysis

**DOI:** 10.2147/DMSO.S458238

**Published:** 2024-09-09

**Authors:** Ravi Deepa, Melissa Glenda Lewis, Onno Van Schayck, Giridhara R Babu

**Affiliations:** 1 Indian Institute of Public Health-Bangalore, Public Health Foundation of India (PHFI), Bengaluru, Karnataka, India; 2 Department of Family Medicine, Care and Public Health Research Institute (CAPHRI), Maastricht University, Maastricht, Limburg, the Netherlands; 3 Indian Council of Medical Research-National Institute of Cholera and Enteric Diseases (NICED), Kolkata, India; 4 Department of Population Medicine, College of Medicine, QU Health, Qatar University, Doha, Qatar

**Keywords:** gestational diabetes, hyperglycaemia, caesarean section, adiposity, obesity

## Abstract

**Background:**

To assess the association of adverse pregnancy and infant outcomes with different cut-off levels of glucose intolerance during pregnancy in the MAASTHI cohort.

**Design:**

Pregnant women (n = 1470) underwent Oral glucose tolerance test between 24 and 36 weeks using a 75-g oral glucose load, with plasma glucose estimations measured at fasting and two hours later. Follow-up was done within 72 hours of delivery for recording type of delivery, infant weight, mid-upper arm circumference, and skinfold thickness.

**Results:**

The odds of having higher skinfold thickness (>90th percentile) were 43% higher (AOR = 1.43; 95% CI: 1.18, 1.74) and the odds of being overweight at birth was 34% higher (AOR = 1.34; 95% CI: 1.09, 1.62) for every 1 standard deviation (9.9 mg/dL) increase in fasting plasma glucose (FPG) in male infants. The odds of delivering via caesarean section were 45% higher in women with female foetus (1.45,95% CI 1.15,1.82) for every one SD (23.4 mg/dl) increase in 2-h post-load Glucose.

**Conclusion:**

The impact of maternal glucose levels on infant and maternal outcomes differed notably between sex of the child. Compared to female infants, male infants exhibited a stronger association with elevated risks for adverse outcomes, including higher infant weight and increased skinfold thickness.

## Plain Language Summary

Glucose intolerance, in simple terms, refers to a condition where the body has difficulty processing sugar (glucose) properly. Normally, when we eat, our body breaks down carbohydrates into glucose, which is then used by cells for energy. However, in glucose intolerance, this process does not work as efficiently. This can lead to higher-than-normal blood sugar levels, which, if persistent, can increase the risk of developing type 2 diabetes over time. In this study, the researchers investigated how glucose intolerance during pregnancy results in negative health outcomes in mothers and infants in a South Indian City. This is significant as the adverse impact of glucose intolerance in Indian women is not widely studied. The Hyperglycemia and Adverse Pregnancy Outcome (HAPO) study was considered by WHO for defining GDM diagnosis primarily focused on Caucasian populations. Given the known ethnic differences in glucose metabolism and the high prevalence of gestational diabetes in India, understanding glucose intolerance, specifically in Indian women, is essential. In this research, all pregnant mothers underwent an oral glucose tolerance test between the 24th and 36th weeks of pregnancy. They fasted for 12 hours before their blood samples were taken to measure their fasting glucose levels. Then, they drank a glucose solution containing 75 g of glucose. After waiting for 2 hours, their glucose levels were measured again. Those with fasting glucose levels equal to or greater than 92 mg/dl and 2-hour post-load plasma glucose levels equal to or greater than 153 mg/dl were diagnosed with gestational diabetes mellitus (GDM). After delivery, the weight and fat deposition under the skin; known as skinfold thickness (adiposity) were measured using a skinfold caliper.

The risks of higher weight and skinfold were seen in male infants compared to female infants. The overall risk of adiposity and C-section were higher than those reported in the HAPO study, highlighting the need for large-scale studies among the Indian population to better understand and address these associations.

## Introduction

The epidemic of obesity and its associated health complications continues to pose a major public health challenge worldwide, necessitating a comprehensive understanding of its early determinants. Infant adiposity, marked by an excess accumulation of body fat, has been identified as a critical contributor to the development of metabolic and cardiovascular diseases later in life. The significance of the intrauterine environment, profoundly influenced by maternal metabolic health, is undeniably crucial in determining the neonate’s future health trajectory. Gestational Diabetes Mellitus (GDM) is the onset or first recognition of any degree of glucose intolerance during pregnancy and is the most prevalent metabolic disorder among expectant mothers.^1^ The International Diabetes Federation reported pooled global standardized GDM prevalence to be 14.0% (95% confidence interval: 13.97–14.04%) and one in five women in South East Asia are reported to be diagnosed with GDM.^2^ The prevalence estimates of GDM in India show significant diversity, ranging from 0 to almost 41.9%.^3^

During pregnancy, impaired glucose tolerance leads to foetal growth stimulation resulting in macrosomia, further resulting in delivery problems like shoulder dystocia, birth injuries, and intrauterine foetal deaths.^4^ The foetal macrosomia also results in perineal tears and necessitates delivering by caesarean section. In the mother, untreated elevated glucose levels can lead to the development of type 2 diabetes, renal morbidity, and cardiovascular diseases (CVD).^5–9^ Evidence is mounting to suggest that maternal hyperglycemia, even in the absence of gestational diabetes mellitus (GDM), is associated with adverse pregnancy outcomes, including heightened risks of infant adiposity and caesarean section (C-section).^10^,^11^ This underlines the urgent need to elucidate the relationships between maternal glucose levels, mode of delivery, and infant adiposity to inform timely interventions and risk reduction strategies.

There is controversy regarding the glucose intolerance level, which is clinically significant for achieving optimal health outcomes for both mother and child. Diagnosis of GDM is flawed with diverse diagnostic criteria and varying cut-off values across the globe.^12^ This is particularly problematic in countries with limited evidence regarding the consequences of varying glucose levels, especially in the sub-threshold beneath the conventional cut-off values. Hyperglycemia and Adverse Pregnancy Outcome (HAPO) study showed that the relationship of plasma glucose to adverse events is continuous, followed by which International Association of Diabetes and Pregnancy Study Group (IADPSG) set criteria for diagnosing GDM in 2008,^13^ where the threshold for fasting plasma glucose is ≥ 92 mg/dl, cut-off for one-hour plasma glucose is ≥180 mg/dl, and two-hour plasma glucose is ≥ 153 mg/dl.^14^ Asian Indians have higher 2-h plasma glucose levels than Caucasians due to their greater insulin resistance. Despite HAPO including Asians, greater and updated contextual evidence is required due to the high prevalence of GDM and varied risk profiles in the region.^15^

Despite the global recognition of the adverse maternal and fetal outcomes associated with glucose values below the cut-off values, a significant gap exists in defining the specific maternal glucose levels that correlate with increased risks of infant adiposity and C-section, particularly in the context of diverse populations in India. Therefore, we hypothesize that glucose levels below IADPSG recommended levels are associated with adverse pregnancy and infant outcomes like overweight, adiposity, preterm delivery, and caesarean section. We aimed to explore the associations of adverse pregnancy and infant outcomes with different cut-off levels of glucose intolerance during pregnancy by using generalized additive models (GAM) and Quantile methods in the Maternal antecedents of adiposity and studying the transgenerational role of hyperglycaemia and insulin (MAASTHI) birth cohort in South India.

## Material and Methods

MAASTHI is a prospective cohort study evaluating the link between maternal hyperglycaemia and child outcomes in the first five years of life. The objective of the MAASTHI study was to investigate the effect of glucose levels in pregnancy on skinfold thickness (adiposity) in infancy as a marker of future obesity and diabetes in offspring. A detailed cohort protocol with study design and methodology was published earlier.^16^ MAASTHI enrolled pregnant women with a singleton pregnancy between 24- and 36 weeks’ gestation, they were screened for Gestational diabetes through the IADPSG-diagnostic criteria for Oral Glucose Tolerance Test (OGTT) ie ≥92 mg/dl (≥5.2 mmol/l) or 1-hour ≥ 180 mg/dl (≥10 mmol/l) or 2-hour ≥ 153 mg/dl (≥8.5 mmol/l); however, we did not perform 1 hour PG test due to feasibility issues. Women who voluntarily agreed to participate and provided written informed consent were recruited into the study. Women with chronic conditions/illnesses were excluded from the study. The ethics committee approval was obtained from the Institutional Ethics Committee at the Indian Institute of Public Health – Bengaluru.

### Exposure

All pregnant women underwent an oral glucose tolerance test between 24 and 36 weeks using a 75-g oral glucose load, with plasma glucose estimations taken at fasting and two hours later. All women with a fasting plasma glucose concentration greater than or equal to 92 mg/dl and a 2-h post-load Plasma Glucose (2-h PG) greater than or equal to 153 mg/dl were diagnosed with gestational diabetes mellitus (GDM).

### Outcome

Infant size at birth was the primary outcome of interest. At birth, follow-up was conducted within 72 hours of delivery for recording type of delivery, infant weight, mid-upper arm circumference (MUAC), and measurements of skinfold thickness at biceps, triceps, and sub-scapular regions. Skinfolds were measured to the nearest 2 mm with a calliper (Holtain T/W Skinfold Caliper, Holtain, Crymych, UK). The sum of skinfold thickness (SSFT) was calculated by adding together the average measurements from the bicep, triceps, and subscapular regions.

Trained research assistants entered all data into a validated Android application specifically designed for the cohort. For this study, records of 1470 women whose children’s anthropometry were measured at birth were included.

### Statistical Analysis

Categorical variables are reported using numbers and percentages, whereas continuous variables are presented using Mean ± Standard deviation (SD) or Median (Interquartile range (IQR)) for normally distributed and skewed variables, respectively. Univariate analysis (independent sample *t*-test and Chi-square test of association) was used to determine the relationship between maternal and infant characteristics with GDM using currently available guidelines for plasma glucose concentrations. Three binary infant outcomes: weight ≥ 90th percentile (3.3 Kg), MUAC ≥ 90th percentile (≥11 cm), Sum of Skinfold Thickness (SSFT) ≥ 90th percentile (≥16.2 mm), and the primary C section as the pregnancy outcome (excluding cases with previous C-section and those who report Placenta Previa and Mal-presentation). For associations with pregnancy and infant outcomes, plasma glucose levels were considered both continuous and categorical in a multivariable logistic regression analysis. Women who were prescribed medication, insulin, and lifestyle modification were excluded from the analysis. For continuous-variable analyses, odds ratios with 95% confidence intervals (CI) were calculated for a one SD increase in plasma glucose levels for female and male infants. The Quantile Regression (QR) was performed on the “birth weight” variable at different quantiles ranging from 0.1 to 0.9. QR was performed to establish two models to explore how different infant weight levels were associated with FPG/2-h PG (model 1 included univariate analysis and model 2 was adjusted for age, mother’s height, socioeconomic status, husbands’ income in rupees, family history of diabetes, gravida, parity, gestational age at the time of OGTT, and gestational age at the time of delivery).

Sensitivity analysis was also performed for all the infant and pregnancy outcomes using Generalized Additive Models (GAM) to understand the non-linearity terms. The amount of non-linearity of the smooth function was assessed using the effective degrees of freedom (EDF). Logistic regression was used to explore the relationships between the optimal glucose classification as per the HAPO study and adverse pregnancy outcomes for both female infants and male infants. The logistic regression and GAM models were adjusted with variables, namely age in years, height in centimetres, socioeconomic status, husbands’ income in rupees, family history of diabetes, gravida, parity, gestational age at the time of OGTT, and gestational age at the time of delivery. R version 4.3.1 (University of Auckland, Oakland, New Zealand) was used to perform the statistical analyses. Statistical significance was set at P-value < 0.05.

## Results

**Figure 1:**
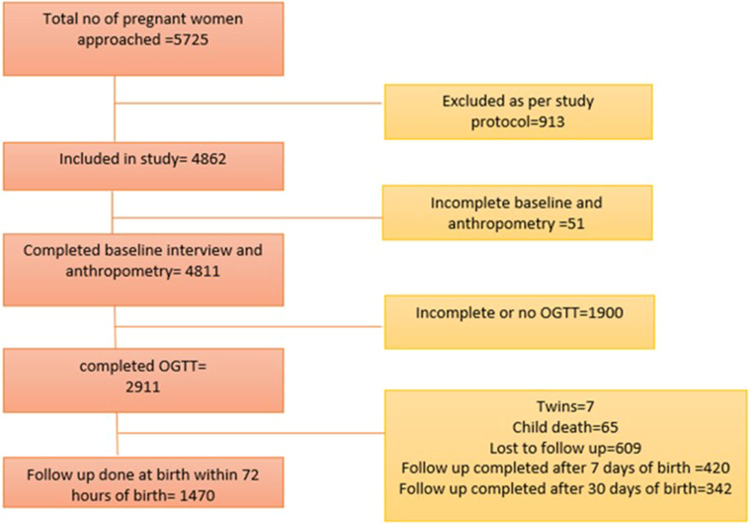
Study flowchart depicting participant recruitment.

A total of 1470 mother-infant pairs were included in this study (Figure 1). The baseline characteristics of the pregnant women and infants are provided in Table 1. The mean age of the women was 24.3 years, with a gestational age of 24.3 weeks (at the time of recruitment) and 28.3 weeks at the time of OGTT. Nearly half (n = 729, 49.6%) of the women belonged to the Hindu religion, about n = 863, 58.7% belonged to the lower socioeconomic class, with the majority being unemployed (n = 1370, 93.2%), more than three-fifths, n = 913, 62.1% were multigravida. More than one-fifth (n = 323, 22%) of the women had a family history of diabetes mellitus. About n = 209, 14.2% had gestational diabetes mellitus at baseline, and one-third of the women n = 430, 29.3% had primary caesarean section delivery. The mean FPG and 2-h PG levels were 82.1 mg/dl and 108.4 mg/dl, respectively. Nearly one-tenth (n = 133, 9%) of the infants were born premature (before 37 weeks of gestation). There was an equal distribution of male and female infants in the study. Of the total 1470 infants, the mean MUAC, sum of skinfold thickness, and weight were 9.7 cm, 4.6 mm, and 2.8 kg, respectively.

**Table 1:** Baseline Characteristics of Pregnant Women and Infant Outcomes (N = 1470)

Characteristic or Outcome	Categories	N (%)
Age in Years	Mean ±SD	24.28 ± 4.05
Height in centimetres	Mean ±SD	154.22 ± 5.66
Gestational Age at the time of Baseline Interview in weeks	Mean ±SD	24.27 ± 5.49
Gestational age at the time of OGTT in weeks	Mean ±SD	28.31 ± 3.14
Religion	Hinduism	729 (49.6%)
	Islam	685 (46.6%)
	Christianity	54 (3.7%)
	Others^#^	2 (0.1%)
Husbands Income in Rupees	Median (IQR)	10000 (9000, 15,000)
Socio economic status	Lower class	863(58.7%)
	Middle class	457(31.1%)
	Upper class	150(10.2%)
Employment	Unemployed	1370(93.2%)
	Employed	100 (6.8%)
Parity	Nulliparous	632(43%)
	Primiparous	696(47.3%)
	Multiparous	142(9.7%)
Gravida	Primigravida	557(37.9%)
	Multigravida	913(62.1%)
Fasting plasma glucose (mg/dl)	Mean ±SD	82.09±9.87
2-h postload plasma glucose (mg/dl)	Mean ±SD	108.39±23.47
Family history of diabetes mellitus	Yes	323(22%)
	No	1147(78%)
Gestational diabetes mellitus at baseline	Yes	209(14.2%)
	No	1261(85.8%)
MUA Circumference in cm	Mean ±SD	26.10±3.76
Sum of skinfold thickness in mm	Mean ±SD	17.11±5.87
Primary Caesarean Section	Yes	430(29.3%)
	No	1040(70.7%)
Gestational age at the time of delivery in weeks	≥37 weeks	1337(91%)
	<37 weeks	133(9%)
**Infant Characteristics**		
Gender	Male	745(50.7%)
	Female	725(49.3%)
Resuscitation	Yes	926(63%)
	No	544(37%)
Aspirate	Yes	533(36.3%)
	No	937(63.7%)
Morbidity	Yes	112(7.6%)
	No	1358(92.4%)
MUA Circumference in cm	Mean ±SD	9.66±0.97
Sum of skinfold thickness in mm	Mean ±SD	4.57±1.01
Weight in kilograms	Mean ±SD	2.78±0.40

**Notes**: *# Religion other category included Jainism and no religion.*

The relationship between maternal and infant characteristics with GDM (FPG ≥ 92 and/or 2-h PG ≥ 153) is provided in Table 2 separately for male and female infants. MUAC > 90th percentile (11 cm), the sum of skinfold thickness >90th percentile (16.2 mm), and weight >90th percentile (3.3 Kg) were significantly associated with GDM in male infants and not female infants.

**Table 2:** Maternal and Infant Characteristics and Its Relationship with Gestational Diabetes in Male and Female Infants (N = 1470)

Characteristic or Outcome	Categories	All			Boys			Girls		
		GDM (n=209)	No GDM (n=1261)	P value	GDM (n=121)	No GDM (n=624)	P value	GDM (n=88)	No GDM (n=637)	P value
		N (%)	N (%)		N (%)	N (%)		N (%)	N (%)	
**Maternal characteristics**										
Age in years^†^	Mean ± SD	25.33±4.20	24.11±4.01	<0.001*	24.06±4.13	25.17±4.06	0.007	25.56±4.38	24.15±3.88	0.002
Height in centimetres^†^	Mean ± SD	154.79±5.30	154.12 ±5.72	0.11	154.7±5.31	154.1±5.85	0.30	154.92±5.29	154.1±5.57	0.21
Gestational Age at the time of Baseline Interview in years (in weeks)^†^	Mean ± SD	24.30±5.41	24.26±5.50	0.11	24.21±5.13	24.23±5.64	0.97	24.41±5.79	24.29±5.36	0.84
Gestational age at the time of OGTT (in weeks)^†^	Mean ± SD	28.37±2.74	28.30 ±3.20	0.709	28.19±2.60	28.25±3.23	0.83	28.63±2.90	28.34±3.17	0.41
Parity	Nulliparous	79(37.80%)	553(43.85%)	0.19	46(38.0%)	285(45.7%)	0.21	33(37.5%)	268(42.1%)	0.007
	Primiparous	111(53.11%)	585(46.39%)		65(53.7%)	281(45.0%)		46(52.3%)	304(47.7%)	
	Multiparous	19(9.09%)	123(9.75%)		10(8.3%)	58(9.3%)		9(10.2%)	65(10.2%)	
Gravida	Primigravida	65(31.10%)	492(39.02%)	0.03*	83(68.6%)	370(59.3%)	0.05	61(69.3%)	399(62.6%)	0.22
	Multigravida	144(68.90%)	769(60.98%)		38(31.4%)	254(40.7%)		27(30.7%)	238(37.4%)	
Socioeconomic status	Upper lower class	127(60.77%)	736(58.37%)	0.72	72(59.5%)	374(60%)	0.78	55(62.5%)	362(56.8%)	0.71
	Lower middle class	60(28.71%)	397(31.48%)		34(28.1%)	192(30.8%)		26(29.5%)	205(32.2%)	
	Upper class	22(10.53%)	128(10.15%)		15(12.4%)	58(9.2%)		7(8.0%)	70(11%0	
Sum of skinfold thickness (mm)	≥90^th^ percentile (66 mm)	48(22.97%)	101(8.01%)	<0.001*	32(26.4%)	60(9.6%)	0.00	14(15.9%)	42(6.6%)	0.002
	<90^th^ percentile	161(77.03%)	1160(91.99%)		89(73.6%)	564(90.4%)		74(84.1%)	595(93.4%)	
MUA Circumference (cm)	≥90^th^ percentile (31 cm)	49(23.44%)	108(8.56%)	<0.001*	22(18.2%)	54(8.7%)	0.002	15(17.0%)	65(10.2%)	0.05
	<90^th^ percentile	160(76.56%)	1153(91.44%)		99(81.8%)	570(91.3%)		73(83.0%)	572(89.8%)	
Primary Caesarean Section	Yes	61(29.2%)	279(22.1%)	0.02	39(32.2%)	129(20.7%)	0.005	22(25.0%)	150(23.5%)	0.76
	No	148 (70.8%)	982(77.9%)		82(67.8%)	495(79.3%)		66(75.0%)	487(76.5%)	
Gestational age at the time of delivery (in weeks)	≥37 weeks	189(90.43%)	1148(91.04%)	0.78	110(90.9%)	53(8.5%)	0.83	79(89.8%)	60(9.4%)	0.80
	<37 weeks	20(9.57%)	113(8.96%)		11(9.1%)	571(91.5%)		9(10.2%)	577(90.6%)	
Infant Characteristics										
Cried after Birth	Yes	202(96.65%)	1225(97.15%)	0.69	116(95.9%)	609(97.6%)	0.28	86(97.7%)	615(96.5%)	0.56
	No	7(3.35%)	36(2.85%)		5(4.1%)	15(2.4%)		2(2.3%)	22(3.5%)	
Resuscitation	Yes	142(67.94%)	784(62.17%)	0.11	44(36.4%)	259(41.5%)	0.29	30(34.1%)	253(39.7%)	0.31
	No	67(32.06%)	477(37.83%)		77(63.6%)	365(58.5%0		58(65.9%)	384(60.3%)	
Aspirate	Yes	85(40.67%)	448(35.53%)	0.15	2(1.7%)	21(3.4%)	0.31	3(3.4%)	28(4.4%)	0.66
	No	124(59.33%)	813(64.47%)		119 (98.3%)	603 (96.6%)		85(95.6%)	609(95.6%)	
Weight in Kgs†	Mean ± SD	2.88(0.43)	2.76(0.39)	<0.001*	2.89(0.45)	2.79(0.38)	0.012	2.88(0.39)	2.73(0.39)	0.002
Weight in Kgs	≥90^th^ percentile (3.3 kg)	33(15.79%)	118(9.36%)	<0.005*	23(19.0%)	68(10.9%)	0.01	10(11.4)	50(7.8)	0.26
	<90^th^ percentile	176(84.21%)	1143(90.64%)		98(81.0%)	556(89.1%)		78(88.6%)	587(92.2)	
MUA Circumference	≥90^th^ percentile (11 cm)	37(17.70%)	119(9.44%)	<0.001*	22(18.2%)	54(8.7%)	0.002	15(17.0%)	65(10.2%)	0.05
	<90^th^ percentile	172(82.30%)	1142(90.56%)		99(81.8%)	570(91.3%)		73(83.0%)	572(89.8%)	
Sum of skinfold thickness	≥90^th^ percentile (16.2 mm)	42(20.10%)	105(8.33%)	<0.001*	35 (28.9%)	85 (13.6%)	0.00	17 (19.3%)	81 (12.7%)	0.09
	<90^th^ percentile	167(79.90%)	1156(91.67%)		86 (71.1%)	539 (86.4%)		71 (80.7)	556 (87.3%)	

**Notes**: # FPG≥92 and/or 2-h PG ≥ 153 classified as GDM. †Independent sample *t*-test instead of Chi-square test of association. *p=<0.05.

Among the 209 women diagnosed with GDM, most did not receive any treatment (n = 129), while information on treatment was unavailable for 17 women, approximately one-fifth received physician-recommended diet and physical activity guidelines (n = 56), and a small percentage were prescribed insulin or oral medications (n = 7). Women who confirmed receiving any treatment or adhering to the doctor-prescribed diet and lifestyle modifications were excluded from the analysis. (Not mentioned in Table)

Table 3 provides the analysis of the FPG and 2-h PG levels as a continuous variable where the odds ratios are presented for every 1 SD increase in the plasma glucose levels (excluding those women who received treatment/lifestyle modification recommendation by doctor). One SD FPG and 2-h PG are equivalent to 9.9 mg/dL glucose and 21.6 mg/dL glucose, respectively. Among male infants, for every 1 SD increase in FPG, the odds of the infant’s weight being ≥ 90th percentile increases by 34% and the odds of skinfold thickness ≥ 90th percentile increases by 43%. FPG and 2-h PG are not significantly associated with weight in female infants, except for subscapular skinfold thickness ≥ 90th percentile 1.35 (1.08,1.69) and 1.36 (1.08,1.72), respectively.

**Table 3:** Adjusted Odds Ratios for Associations Between Maternal Glucose as a Continuous Variable and Pregnancy, Infant Outcomes (n = 1445)

		HAPO	MAASTHI			
			Male Infants		Female Infants	
Outcome	Exposure	AOR (95% CI)	AOR (95% CI)	p value	AOR (95% CI)	p value
**Infant weight ≥90^th^ percentile**	**Fasting**	1.38 (1.32, 1.46)	1.34 (1.09, 1.62)	0.004	1.05 (0.78, 1.42)	0.73
	**2-h- PG**	1.38 (1.32, 1.46)	1.42 (1.11,1.82)	0.005	1.30 (0.98,1.72)	0.06
**Infant Triceps of skin fold ≥90^th^ percentile**	**Fasting**	1.40 (1.34, 1.48)	1.64 (1.32, 2.03)	<0.001	1.04 (0.81, 1.35)	0.71
	**2-h- PG**	1.38 (1.31, 1.45)	1.76 (1.37,2.2)	<0.001	1.11 (0.86,1.43)	0.40
**Infant Subscapular of skin fold ≥90^th^ percentile**	**Fasting**	1.43 (1.36, 1.51)	1.40 (1.16, 1.70)	<0.001	1.35 (1.08,1.69)	0.007
	**2-h- PG**	1.37 (1.30, 1.43)	1.29 (1.02,1.63)	0.03	1.36 (1.08,1.72)	0.009
**Infant Sum of skin fold ≥90^th^ percentile**	**Fasting**	NA	1.43 (1.18, 1.74)	<0.001	1.24 (0.98, 1.56)	0.06
	**2-h- PG**	NA	1.61 (1.28,2.02)	<0.001	1.22 (0.96,1.55)	0.09
**Infant MUAC ≥90^th^ percentile**	**Fasting**	NA	1.13 (1.07, 1.67)	0.009	1.34 (0.87,1.46)	0.331
	**2-h- PG**	NA	1.47 (1.11,1.93)	0.006	1.36 (1.05,1.76)	0.01
**Primary caesarean section**	**Fasting**	1.11 (1.06, 1.15)	1.15 (0.96, 1.38)	0.122	1.21 (0.97,1.50)	0.08
	**2-h- PG**	1.08 (1.03, 1.12)	1.22 (0.99,1.51)	0.05	1.45 (1.15,1.82)	0.001

Associations were adjusted for the following variables: age in years, height in centimetres, socioeconomic status, husbands’ income in rupees, family history of diabetes, gravida, parity, gestational age at OGTT, gestational age at the time of delivery and child gender. Excluded women who were on treatment; insulin, metformin and were counselled for change in diet and physical activity levels.

For every one standard deviation increase in 2-hour postprandial glucose (2-h PG) levels there is a 42% increased odds of the weight being ≥ 90th percentile, a 61% increased odds of the sum of skinfold thickness being ≥ 90th percentile, and a 47% higher odds of MUAC being ≥ 90th percentile in male infants. The association of 2-h PG with weight ≥ 90th percentile shows borderline significance in female infants; however, there was a 45% greater odds of delivering via Caesarean section for one SD increase in 2-h PG.

For every 1 SD increase in FPG (9.87 mg/dl) and 2-h PG (23.47), the odds of triceps being greater than the 90th percentile was 1.64 (1.32, 2.03) and 1.76 (1.37,2.2) in male infants, this is much higher than HAPO study that reported the odds of 1.40 (1.34, 1.48) for 1 SD increase in FPG (7.20 mg/dl) and 1.38 (1.31–1.45) for 1 SD increase in 2-h PG (23.4 mg/dl).^17^ It is important to note that again female infants in our study had a much lower odds of triceps adiposity compared to male infants.

**Figure 2:**
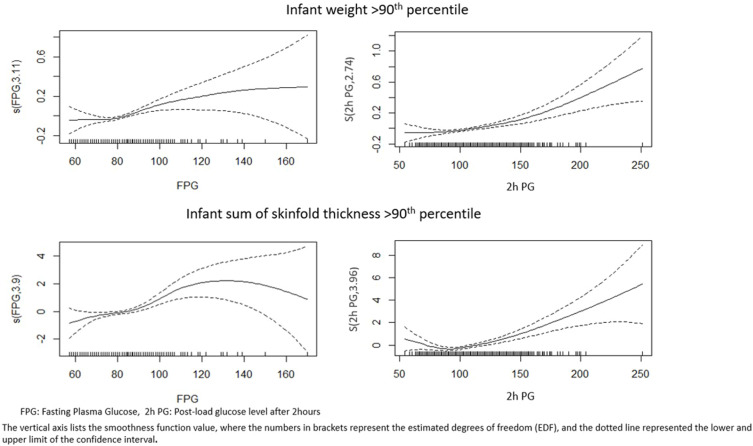
Generalized additive model (GAM) plots showing the partial effects of plasma with infant weight ≥90th percentile and sum of skinfold thickness ≥90th percentile.

**Figure 3:**
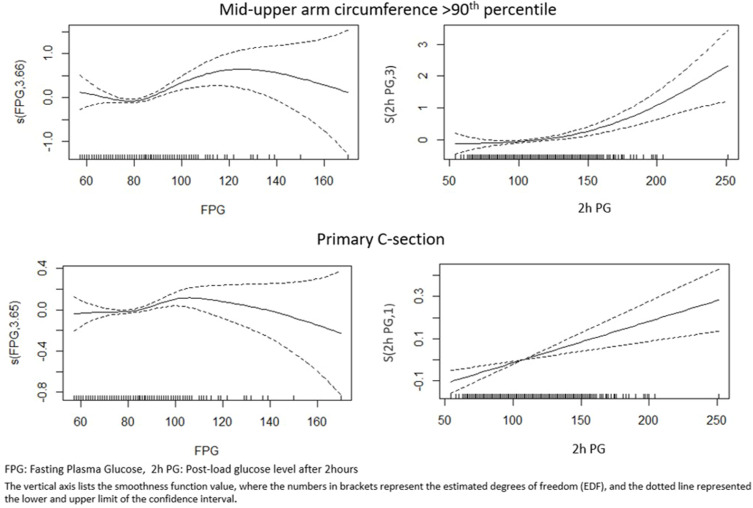
Generalized additive model (GAM) plots showing the partial effects of plasma with MUAC ≥90th percentile and primary C-section delivery.

The results of GAM models are provided in Figures 2 and 3 and the summary statistics are provided in Supplementary Table 1. Generalised Additive Model results provide insights into the relationships between the outcomes a) Infant weight ≥90th percentile b) Infant sum of skinfold thickness ≥90th percentile c) Infant MUAC ≥ 90th percentile d) Primary C section and the smooth functions of FPG and 2-h PG, while considering the adjustment variables age in years, height in centimetres, socioeconomic status, husbands’ income in rupees, family history of diabetes, gravida, parity, gestational age at the time of OGTT, and gestational age at the time of delivery. The graphs show an increase in infant weight with an increase in FPG and 2-h PG values, with significant p values. Consistent rise in trend is seen after 90 mg/dl of FPG, similarly for 2-h PG rise is more pronounced after 150 mg/dl (Figures 2 and 3). Supplementary Table 1 provides the EDF for the relationship between glucose values and infant outcomes, and it shows a linear relationship between 2-h-PG and C-section delivery.

Supplementary Table 2 shows the logistic regression analysis based on cut-offs used in the HAPO study analysis. The number of cases in category 5 to 7 were too low for both FPG and 2-h-PG to make any valid interpretation. Those with maternal FPG between 80 and 84 mg/dl were twice as likely to have birth weight more than 90th percentile among male infants and female infants. The adjusted OR increased with each higher category of FPG for male infants for MUAC > 90th percentile and Skinfold thickness > 90th percentile.

Results of the Quantile Regression model of FPG and 2-h PG with infant weight. Quantile regression (QR) was conducted to estimate how the relationship between “FPG/2h PG” and “weight” varies across different quantiles of the “birth weight” distribution.

The coefficients of FPG in different quantiles of infant weight are shown separately in Table 4. There were positive associations of infant weight with FPG, and the coefficients increased from P10 to P90 (model 1). In addition, FPG was positively associated with weight after adjusting for age in years, socioeconomic status, husbands’ income in rupees, family history of diabetes, gravida, parity, gestational age at the time of OGTT, and gestational age at the time of delivery (model 2). For a one-unit change in FPG and 2-h PG there is 0.007 g and 0.003 g increase in 90th percentile weight, respectively, after adjusting for confounders.

**Table 4:** Quantile Regression Coefficients [95% CI] Between FPG, 2-h PG, and Infant Weight

FPG percentile	P10	P20	P30	P40	P50	P60	P70	P80	P90
Model 1 (Univariate)	0.004* (0.001,0.007)	0.004*(0.0005, 0.006)	0.004* (0.001, 0.007)	0.004* (0.001, 0.)	0.004* (0.001, 0.007)	0.005*(0.002, 0.007)	0.006* (0.002, 0.01)	0.0076* (0.004, 0.01)	0.0077* (0.002, 0.01)
Model 2	0.004* (0.004, 0.01)	0.004* (0.004, 0.01)	0.004* (0.004, 0.01)	0.004* (0.004, 0.01)	0.003* (2.16, 2.65)	0.004* (0.004, 0.01)	0.004* (0.004, 0.01)	0.007* (0.004, 0.01)	0.007* (0.002, 0.01)
**2-h PG percentile**	**P10**	**P20**	**P30**	**P40**	**P50**	**P60**	**P70**	**P80**	**P90**
Model 1 (Univariate)	0.001* (9.42, 0.002)	0.001* (9.42, 0.002)	0.001* (1.14, 0.002)	0.001* (0.0003,0.002)	0.001* (0.0004, 0.002)	0.002* (0.0005,0.003)	0.001* (0.0003, 0.003)	0.0028* (0.004, 0.01)	0.0038* (0.0009, 0.005)
Model 2	0.002* (2.05, 2.25)	0.002* (2.18, 2.50)	0.001* (2.33, 2.59)	0.001* (2.42, 2.61)	0.001* (2.50, 2.73)	0.001* (2.52, 2.80)	0.001* (2.58, 2.91)	0.002* (2.58, 3.10)	0.0034* (2.69, 3.17)

* P <0.05 CI: confidence interval. Model 1, without adjustments for the confounding factors. Model 2 adjusted for age in years, socioeconomic status, husbands’ income in rupees, family history of Diabetes, gravida, parity, gestational age at the time of OGTT, and gestational age at the time of delivery.

## Discussion

The intricate relationship between maternal glucose levels and infant adiposity has been the subject of extensive research, aiming to unravel the potential pathways and implications of metabolic health from pregnancy to early childhood. We show that maternal blood glucose levels are positively associated with infant birth weight and adiposity. By analysing data for male and female infants separately, we could show the variability in infant adiposity among the two sexes.

Male infants showed higher odds of increased birth weight compared to female infants. This is similar to the findings from the HAPO study, although HAPO did not stratify based on the infant’s sex. The MAASTHI male infant had higher triceps skinfold > 90th percentile and the values were much higher than the HAPO study (1.64 v/s 1.40). The data indicate that male infants are more susceptible to adverse effects of elevated maternal glucose levels, highlighting the need for targeted interventions to manage glucose levels during pregnancy. Again, for female infants the odds were much lower, indicating male infants to be at greater risk of adiposity with increasing maternal glucose levels than female infants. Lingwood et al found that in male infants, percent of fat analysed through Air displacement plethysmography was increased by 0.44% for each 0.1 mmol/L increase in mean maternal FPG. They also reported that maternal BMI and non maternal glucose level were the primary predictors of adiposity in female infants.^18^ A study by Benhalima et al reported that gestational weight gain was positively associated with adiposity in male infants only.^12^

In MAASTHI, we found that the sum of skinfold for every 1 SD increase in FPG and 2-h PG was 1.43 (1.18, 1.74) and 1.61 (1.28,2.02), respectively, in males which is significantly greater than that of female infants. A multiethnic cohort in Singapore comprising Chinese, Malay, and Indian populations reported that 1 SD increase in FPG (9.0 mg/dl) and 2-h PG (28.8 mg/dl) was associated with an increase in odds ratios for the sum of skinfold greater than the 90th percentile at 1.64 (95% CI 1.32–2.03) and 1.40 (95% CI 1.10–1.79), respectively. They also note that compared to Chinese, Indians with high fasting glucose levels (>158 mg/dl) are associated with a lesser increase in the sum of skinfold thickness.^19^

The South Asian population not only faces a higher risk of maternal hyperglycaemia but also exhibits significant variations within the group. Thus, studies within each country are necessary to determine the adverse impacts and establish recommended cut-off points. Indian babies are known to have greater adiposity even while having a low birth weight, this is reported in two studies that compared them with Caucasian babies born in Southampton babies.^20^,^21^ This thin-fat phenotype is further associated with cardiometabolic risk factors in adult life and hence requires early screening and lifestyle intervention to reduce the risks and improve long-term health outcomes. HAPO demonstrated that ORs ranged between 1.35 (1.28–1.42) to 1.44 (1.37–1.52) for each glucose measure higher by 1 SD.^17^ In Singapore, each 1 SD increase in fasting and postprandial glucose was associated with an increase in odds ratios for the sum of skinfold greater than the 90th centile at 1.64 (95% CI 1.32–2.03) and 1.40 (95% CI 1.10–1.79), respectively. The influence of high maternal fasting glucose on the neonatal sum of skinfold thickness was less pronounced in Indians compared with the Chinese.^19^

Caesarean section rates were higher among GDM mothers (33.9%) than non-GDM mothers (28.4%) in our cohort but were not statistically significant with GDM diagnosis. However, we found that an increase in 1 SD FPG and 2-h PG resulted in a higher odds of being delivered through caesarean section and the odds were higher than that reported by the HAPO study.^17^ It is well established that GDM increases the risk of caesarean delivery, and several studies have provided evidence to support this.^22–24^ Untreated-GDM women have a greater incidence of caesarean section (22.5%) when compared to treated-GDM women (8.5%),^25^ GDM women also have 34% greater costs of care when compared to non-GDM women.^26^ Screening and managing GDM could be one of the ways through which caesarean deliveries and out-of-pocket expenses can be avoided. GDM women who receive treatment on time have no increased need for caesarean section.^27^

In MAASTHI, despite male infants having higher odds of being overweight, female infants had higher odds of being delivered through C-section than males. However, a study in Italy reported contradictory findings where females had lower risk of caesarean sections in primiparous GDM pregnancies.^28^

The recent Indian National Family Health Survey-5 across the country showed that one in five births is through caesarean delivery.^29^ It should also be noted that such high rates of C-sections may not always be due to medical conditions and could be driven by economic reasons too.^30^ Therefore, it is challenging to pinpoint the actual reasons for C-sections and their biological determinants.

In this study, we observed that the odds of triceps adiposity and c-section are higher than those reported in the HAPO study, which formed the basis for the IADPSG recommendations. This variation underscores the need for expanding the contextual evidence base in the Indian population, to assess and minimize the adverse pregnancy outcomes with different glucose levels, as nearly 20% of healthcare professionals have reported adopting the IADPSG guidelines for diagnosing GDM.^31^

The majority of health professionals follow the Indian National Guidelines recommending GDM diagnosis based on a non-fasting 75 g- 2-h Oral glucose challenge test (OGCT) with 2-h PG > 140 mg/dL, and FPG is not recommended considering the feasibility issues. In light of our study findings, not undertaking FPG could be a matter of concern as fasting values above 90 mg/dL have been known to show a greater risk of adverse pregnancy and child outcomes in our study. Another area of concern is that most healthcare practitioners are unaware of the accurate diagnostic criteria, thus making diagnosis difficult. The findings from our study conducted in the MAASTHI birth cohort in South India offer valuable insights into this complex interplay, contributing to the existing body of knowledge and informing future studies that could positively impact clinical practices and policymaking in India. The established positive associations between maternal glucose levels and indicators of infant adiposity reinforce the importance of stringent glucose monitoring and management during pregnancy. The observed associations underscore the potential benefits of early interventions to modulate maternal glucose levels, to reduce the risk of infant adiposity and its associated long-term health implications. Furthermore, our findings contribute to the ongoing discourse surrounding the appropriate diagnostic criteria and cut-off values for gestational diabetes, particularly in the Indian context. The variation in associations observed across different glucose levels highlights the need for context-specific research to establish evidence-based guidelines that are tailored to the unique metabolic profiles of different populations. This is especially pertinent in countries like India, where the prevalence of gestational diabetes is on the rise., and the current diagnostic criteria may not adequately capture the entire range of glucose intolerance and its implications on infant health.

Our study has several limitations. First, glucose data were not collected at 1-hour post-load but only at two-time points: fasting and 2-hour post-glucose load, limiting our understanding of glucose dynamics. Second, adiposity was assessed using skinfold thickness, which provides an indirect measure of body fat. Additionally, we did not have access to pre-pregnancy BMI data. These limitations prevent us from assessing the influence of pre-existing obesity and maternal weight gain on infant outcomes.

## Conclusion

The data presented demonstrate a robust and consistent relationship between maternal blood glucose levels and neonatal weight and adiposity. The impact of maternal glucose levels on infant outcomes differed notably between sexes and the risk was much higher in male infants than female infants. The infants in the MAASTHI cohort were also at higher odds of greater adiposity and C-section than the population described in the HAPO study. The current diagnostic criteria for GDM may not fully capture the spectrum of glucose intolerance and its impact on maternal and neonatal health, especially in the Indian context, thus necessitating the conduct of large-scale studies in the country.

## Data Availability

The datasets used and/or analyzed during the current study are available from the corresponding author on reasonable request.

## References

[1] Care ADAJD. Diagnosis and classification of diabetes mellitus. *Diabet Care*. 2014;37(Supplement_1):S81–S90. 10.2337/dc14-S081 24357215

[2] Wang H, Li N, Chivese T, et al. IDF Diabetes Atlas: estimation of Global and Regional Gestational Diabetes Mellitus Prevalence for 2021 by International Association of Diabetes in Pregnancy Study Group’s Criteria. *Diabet Res Clin Pract*. 2022;183:109050. doi:10.1016/j.diabres.2021.109050 34883186

[3] Li KT, Naik S, Alexander M, Mathad JS. Screening and diagnosis of gestational diabetes in India: a systematic review and meta-analysis. *Acta Diabetologica*. 2018;55(6):613–625. doi:10.1007/s00592-018-1131-1 29582160 PMC5999405

[4] Kwik M, Seeho SKM, Smith C, McElduff A, Morris JM. Outcomes of pregnancies affected by impaired glucose tolerance. *Diabet Res Clin Pract*. 2007;77(2):263–268. doi:10.1016/j.diabres.2006.12.004 17275121

[5] Hu J, Gillies CL, Lin S, et al. Association of maternal lipid profile and gestational diabetes mellitus: a systematic review and meta-analysis of 292 studies and 97,880 women. *EClinMed*. 2021;34:1. 10.1016/j.eclinm.2021.100830 PMC8102708 33997732

[6] Goyal A, Gupta Y, Kalaivani M, et al. Long term (>1 year) postpartum glucose tolerance status among Indian women with history of Gestational Diabetes Mellitus (GDM) diagnosed by IADPSG criteria. *Diabet Res Clin Pract*. 2018;142:154–161. doi:10.1016/j.diabres.2018.05.027 29802954

[7] Retnakaran R, Qi Y, Sermer M, Connelly PW, Hanley AJG, Zinman B. Glucose Intolerance in Pregnancy and Future Risk of Pre-Diabetes or Diabetes. *Diabet Care*. 2008;31(10):2026–2031. doi:10.2337/dc08-0972 PMC2551649 18628572

[8] Retnakaran R, Shah BR. Mild glucose intolerance in pregnancy and risk of cardiovascular disease: a population-based cohort study. *CMAJ: Canadian Medical Association Journal = Journal de l’Association Medicale Canadienne*. 2009;181(6–7):371–376. doi:10.1503/cmaj.090569 PMC2742157 19703913

[9] Gunderson EP, Chiang V, Pletcher MJ, et al. History of gestational diabetes mellitus and future risk of atherosclerosis in mid-life: the Coronary Artery Risk Development in Young Adults study. *J Am Heart Assoc*. 2014;3(2):e000490. doi:10.1161/JAHA.113.000490 24622610 PMC4187501

[10] Nicolosi BF, Vernini JM, Costa RA, et al. Maternal factors associated with hyperglycemia in pregnancy and perinatal outcomes: a Brazilian reference center cohort study. *Diabetol Metab Syndr*. 2020;12(1):49. doi:10.1186/s13098-020-00556-w 32518595 PMC7275406

[11] Kubo A, Ferrara A, Windham GC, et al. Maternal Hyperglycemia During Pregnancy Predicts Adiposity of the Offspring. *Diabet Care*. 2014;37(11):2996–3002. doi:10.2337/dc14-1438 PMC4207207 25150158

[12] Benhalima K, De Landtsheer A, Van Crombrugge P, et al. Predictors of neonatal adiposity and associations by fetal sex in women with gestational diabetes mellitus and normal glucose-tolerant women. *Acta Diabetologica*. 2021;58(3):341–354. doi:10.1007/s00592-020-01619-0 33216207

[13] International Association of Diabetes and Pregnancy Study Groups Consensus Panel; International Association of Diabetes and Pregnancy Study Groups Recommendations on the Diagnosis and Classification of Hyperglycemia in Pregnancy. *Diabetes Care*. 2010,March 1;33(3):676–682. doi:10.2337/dc09-1848 20190296 PMC2827530

[14] Lowe LP, Metzger BE, Dyer AR, et al. Hyperglycemia and Adverse Pregnancy Outcome (HAPO) Study: associations of maternal A1C and glucose with pregnancy outcomes. *Diabet Care*. 2012;35(3):574–580. doi:10.2337/dc11-1687 PMC3322718 22301123

[15] Gujral UP, Mohan V, Pradeepa R, et al. Ethnic variations in diabetes and prediabetes prevalence and the roles of insulin resistance and β-cell function: the CARRS and NHANES studies. *J Clin Transl Endocrinol*. 2016;4:19–27. doi:10.1016/j.jcte.2016.02.004 27042403 PMC4811044

[16] Babu GR, Murthy GVS, Deepa R, Kumar HK, Karthik M. Maternal antecedents of adiposity and studying the transgenerational role of hyperglycemia and insulin (MAASTHI): a prospective cohort study Protocol of birth cohort at Bangalore, India. *BMC Pregnancy Childbirth*. 2016;16:1–9. doi:10.1186/s12884-015-0735-5 27741952 PMC5065083

[17] Group THSCR. Hyperglycemia and Adverse Pregnancy Outcome (HAPO) Study: associations with neonatal anthropometrics. *Diabetes*. 2009;58(2):453–459. doi:10.2337/db08-1112 19011170 PMC2628620

[18] Lingwood BE, Henry AM, d’Emden MC, et al. Determinants of Body Fat in Infants of Women With Gestational Diabetes Mellitus Differ With Fetal Sex. *Diabet Care*. 2011;34(12):2581–2585. doi:10.2337/dc11-0728 PMC3220854 21994428

[19] Aris IM, Soh SE, Tint MT, et al. Effect of Maternal Glycemia on Neonatal Adiposity in a Multiethnic Asian Birth Cohort. *J Clin Endocrinol Metab*. 2014;99(1):240–247. doi:10.1210/jc.2013-2738 24243635

[20] Yajnik CS, Fall C, Coyaji KJ, et al. Neonatal anthropometry: the thin–fat Indian baby. *Pune Maternal Nutrition Study*. 2003;27(2):173–180. 10.1038/sj.ijo.802219 12586996

[21] Krishnaveni G, Hill J, Veena S, et al. Truncal adiposity is present at birth and in early childhood in South Indian children. *Indian Pediatrics*. 2005;42(6):527. 15995269

[22] Ovesen PG, Jensen DM, Damm P, Rasmussen S, Kesmodel USJTJo M-F, Medicine N. Maternal and neonatal outcomes in pregnancies complicated by gestational diabetes. A nation-wide study. *J Maternal-Fetal Neonatal Med*. 2015;28(14):1720–1724. doi:10.3109/14767058.2014.966677 25228278

[23] Cosson E, Cussac-Pillegand C, Benbara A, et al. Pregnancy adverse outcomes related to pregravid body mass index and gestational weight gain, according to the presence or not of gestational diabetes mellitus: a retrospective observational study. *Diabet Metabol*. 2016;42(1):38–46. doi:10.1016/j.diabet.2015.06.001 26141553

[24] Dahiya K, Sahu J, Dahiya A. Maternal and fetal outcome in gestational diabetes mellitus—a study at tertiary health centre in Northern India. *Open Access Lib J*. 2014;1(03):1.

[25] Wahi P, Dogra V, Jandial K, et al. Prevalence of gestational diabetes mellitus (GDM) and its outcomes in Jammu region. *J Assoc Phys India*. 2011;59(4):227–230. 21755759

[26] Gillespie P, Cullinan J, O’Neill C, Dunne F, Collaborators Ft AD. Modeling the Independent Effects of Gestational Diabetes Mellitus on Maternity Care and Costs. *Diabet Care*. 2013;36(5):1111–1116. doi:10.2337/dc12-0461 PMC3631875 23275358

[27] Bahl S, Dhabhai N, Taneja S, et al. Burden, risk factors and outcomes associated with gestational diabetes in a population-based cohort of pregnant women from North India. *BMC Pregnancy Childbirth*. 2022;22(1):32. doi:10.1186/s12884-022-04389-5 35031013 PMC8759176

[28] Seghieri G, Di Cianni G, Gualdani E, De Bellis A, Franconi F, Francesconi P. The impact of fetal sex on risk factors for gestational diabetes and related adverse pregnancy outcomes. *Acta Diabetologica*. 2022;59(5):633–639. doi:10.1007/s00592-021-01836-1 35037136

[29] International Institute for Population Sciences (IIPS) and ICF., NFHS-5. *National Family Health Survey - 5 2019-21* (Ministry of Health and Family Welfare, Government of India). 2021.

[30] Neuman M, Alcock G, Azad K, et al. Prevalence and determinants of caesarean section in private and public health facilities in underserved South Asian communities: cross-sectional analysis of data from Bangladesh. *India and Nepal*. 2014;4(12):e005982. 10.1136/bmjopen-2014-005982 PMC4283435 25550293

[31] Mahalakshmi MM, Bhavadharini B, Maheswari K, et al. Current practices in the diagnosis and management of gestational diabetes mellitus in India (WINGS-5). *Indian J Endocrinol Metab*. 2016;20(3):364–368. doi:10.4103/2230-8210.180001 27186555 PMC4855966

